# The Cool Kids as a School-Based Universal Prevention and Early Intervention Program for Anxiety: Results of a Pilot Study

**DOI:** 10.3390/ijerph19020941

**Published:** 2022-01-14

**Authors:** Simona Scaini, Federica Rossi, Ronald M. Rapee, Francesca Bonomi, Giovanni M. Ruggiero, Alessia Incerti

**Affiliations:** 1 Child and Youth Lab, Sigmund Freud University, 20143 Milan, Italy; fra.bonomi@outlook.it (F.B.); gm.ruggiero@milano-sfu.it (G.M.R.); 2 Studi Cognitivi Clinics, Center for the Developmental Age, 20136 Milan, Italy; federossi2812@gmail.com; 3 Equipe Kairos, 20061 Milan, Italy; incerti.alessia@gmail.com; 4 Centre for Emotional Health, Department of Psychology, Macquarie University, Sydney, NSW 2109, Australia; ron.rapee@mq.edu.au; 5 Studi Cognitivi, Cognitive Psychotherapy School, 20121 Milan, Italy

**Keywords:** anxiety, children, Cool Kids, CBT, school, intervention, universal program

## Abstract

The efficacy of the Cool Kids program has been consistently demonstrated both within Australia and internationally, but limited data are available on the use of Cool Kids as a universal program. The purpose of the study is to evaluate Cool Kids as a universal program for preventing childhood anxiety in the school context. There were 73 Italian children (35 boys and 36 girls, ages 10–13 years) attending the last year of primary school and the first year of middle school who participated in an active intervention based on a school adaptation of the Cool Kids protocol. Results of *t*-test analyses highlighted a downward trend of anxiety symptoms, especially in total anxiety, somatic anxiety, generalized anxiety, separation anxiety, social anxiety and school phobia at post-treatment assessed by children. Even the score of depression symptoms, measured as a second outcome measure, decreased after the treatment. This study contributes to the evidence base for the Cool Kids program as a universal program for preventing childhood anxiety in the school context. Although these preliminary results show some promise, their replication in future research is necessary given current study limitations.

## 1. Introduction

Anxiety disorders are among the most common psychological disorders in children and adolescents, with current prevalence rates ranging from 4% to 25% [1,2,3]. Anxiety symptoms begin early in life and are often chronic and persistent [4]. Moreover, anxiety disorders in childhood are associated with social, emotional and academic impairment [5,6,7,8], as well as an increased risk for developing other disorders, such as additional anxiety disorders, depression, conduct disorder or substance abuse later in life [9,10,11,12,13,14]. Thus, early recognition and treatment are particularly desirable [15,16,17], especially to prevent the potential for lifelong impact.

Previous reviews and meta-analyses of CBT for anxiety disorders in children and adolescents found moderate to large effect sizes for the effectiveness of interventions [18,19,20,21,22]. Yet, despite the availability of evidence-based programs, anxious children and adolescents rarely receive appropriate treatment for their disorder [23,24]. Less than 20% of young people with clinical anxiety disorders receive help for their disorder [23], and most of this help is unlikely to comprise empirically validated intervention. Further, even when empirically validated treatments are offered, up to 50% of anxious youth continue to meet diagnostic criteria [20]. According to Donovan and Spence (2000) [25], the failure to respond to treatment often occurs when treatment is offered too late and the adverse effects associated with the disorder become ingrained and difficult to reverse.

To circumvent these problems, some authors advocate incorporating interventions into school settings (for a review, see Neil and Christensen, [26]). The school setting has the advantage of being a natural environment that reduces the fear of being “labeled” and it facilitates skills’ generalization [27,28]. School interventions are tailored towards children and teenagers who do not have access to clinical settings or may be reluctant to seek treatment due to the perceived stigma [28]. Indeed, receiving help at school may reduce stigma if seeking help is seen as normal and akin to helping other students with non-psychological difficulties [29,30]. Moreover, schools can provide therapy in an environment that involves real-life challenges for the child. Through collaboration with teachers and student peers, early success on challenging tasks may be maximized [31], facilitating skills acquisition [32]. Moreover, school-based programs can also reduce and alleviate many common barriers to treatment in the community (e.g., time, location, transportation and cost) [33,34]. Although a recent review [35] showed small effect sizes post-intervention for both depression (g = 0.23) and anxiety (g = 0.20) prevention programs delivered in school settings, another previous review [26] indicated that school-based prevention and early intervention programs for anxiety are effective in reducing symptoms of anxiety in children and adolescents, with effect sizes ranging from 0.11 to 1.37. Furthermore, a meta-analysis on social anxiety [22] found that school context studies demonstrated larger effect sizes than clinical setting studies. Indeed, schools provide real-world opportunities to practice facing one’s fears (e.g., social situations, making mistakes, public speaking).

One of the most widely used and empirically validated programs for youth anxiety is the Cool Kids Anxiety Program [36,37]. The Cool Kids program is a manualized CBT program for anxiety disorders in youth (age 7–18 years). Its main components include psychoeducation, cognitive restructuring, child management strategies and graded exposure with additional optional modules addressing social skills, teasing and assertiveness. The efficacy of the Cool Kids program has been consistently demonstrated both within Australia and internationally [38,39,40,41,42]. The program has also been evaluated in a school setting within a low socio-economic population [43]. Results showed that children assigned to active intervention demonstrated a significant reduction in symptoms of anxiety as well as threat-relevant thoughts, relative to children assigned to waitlist and differences, were maintained 4 months after treatment. A subsequent, school-based study [31] found children participating in the active conditions (home-based and school-based interventions) showed greater reductions in anxiety and anxiety-related interference in daily life compared to the waitlist-control group, according to parents’ reports. Finally, a recent trial in Norway delivered through schools to a large sample of adolescents (M age = 14 years) showed significantly greater reductions from Cool Kids relative to the waitlist, and somewhat greater than an active comparison program on symptoms of anxiety and life impairment [44]. 

Delivery of school-based early intervention can follow either universal inclusion (i.e., interventions applied to whole populations, regardless of their risk status) or a targeted focus (selection of “at-risk” sub-populations). Some authors have argued that universal application has several advantages over targeted programs [45]. For example, screening measures used to identify “at risk” children may be subject to false-negative errors [46], leading to the exclusion of children who need assistance. Moreover, selecting “at risk” children may induce social stigma, although some empirical evidence questions this assumption [47]. Therefore, treating children who are already experiencing significant anxiety problems may not be the most effective or efficient means of reducing the incidence of childhood anxiety in the general population. The potential of prevention programs, which intervene prior to the development of significant anxiety symptomatology, needs to be investigated. Recent interest has begun to focus on the adaptation of these programs to the school setting, and a recent meta-analysis showed no statistically significant difference (Q = 0.12, df = 1, *p* = 0.73) in the effect size obtained for universal (g = 0.19, 95% CI: 0.13–0.26) compared to targeted programs (g = 0.22, 95% CI: 0.09–0.34) [35]. 

To date, no studies have investigated the efficacy of Cool Kids as a universal program for preventing childhood anxiety. Previous studies on Cool Kids focused on children affected by anxiety disorders or symptoms, selecting “at-risk” sub-populations. In the current study, the Cool Kids protocol was applied to a whole school population. The program was delivered to all scholars, regardless of the presence or absence of mental health conditions. Thus, unlike the studies on the Cool Kids Program as a selective program, we did not only include children “at-risk” or diagnosed with an anxiety disorder. The sessions helped children develop strategies to handle emotions and establish helpful thought patterns and behaviors. The aim was to evaluate the efficacy of the program in reducing levels of different subtypes of anxiety symptoms. We hypothesized that treated children would show significant improvements in anxiety and coping ability after the intervention. In addition, given the strong relationship between anxiety and depression [48,49,50] suggestions that they share a common underlying diathesis [51], we predicted that treated children would also show reductions in depression.

## 2. Materials and Methods

### 2.1. Participants

A sample of 73 children (mean age: 10.39, age range: 10–13 years old, 35 boys and 36 girls) attending the last year of primary school and the first year of middle school was asked to participate in this research project (89% of the sample attended the Fifth grade and 11% the Sixth grade). All the schools were located in medium-to-large cities in Northern Italy, in areas with mixed socioeconomic backgrounds. Assessing socioeconomic status by Hollingshead’s occupational scale, we obtained the following percentage of score: 0–3: 3.2% mothers, 6.4% fathers; 4–6: 49.1% mothers, 41.3% fathers; 7–9: 47.6% mothers, 52.4% fathers. After receiving a complete description of the study, all the parents and children agreed to participate. Children affected by severe neurological or psychiatric disorders, as declared by parents in a demographic questionnaire, were excluded from the study. All procedures received the approval of the Sigmund Freud University ethics committee and parents signed an informed consent for all participants (protocol code: AAGVFQB@@FUEPA85496, 13 January 2017).

### 2.2. Intervention and Procedure

The adaptation of the Cool Kids protocol to the Italian school context involved a preliminary 2 h meeting with the parents and the teaching staff to illustrate the aims and principles of the intervention. This meeting also involved all operators and adults surrounding the child in practicing some techniques explained in the classroom for the regulation of anxiety, with a view to collaboration to achieve some common goals.

The objectives of the program were:

- Promoting the acquisition of anxiety management skills by the child;
- Increasing theoretical and practical knowledge in the teaching staff on the typical fears in this developmental age and on possible intervention strategies;
- Acting preventively on the child’s psychological well-being to improve adaptation to the school context.

The program was designed to be carried out in five group meetings with the class (in the presence of the teacher), each lasting 2 h, over a period of 7 weeks: the first three meetings were held weekly, while the third and fourth were every two weeks. The program leader was a psychologist who specialized in cognitive-behavioral psychotherapy. 

At the end of the intervention, a meeting for teachers and parents was organized in all the schools.

During the various meetings, the concept of anxiety was illustrated to the children and the main techniques for dealing with it were explained: cognitive restructuring, gradual exposure, problem-solving and “surfing” anxiety. In contrast to the clinical program, the methods used included group work, watching videos and educational games. In addition, compared to the clinical program in which parents played a key role, in our school version of Cool Kids, parents were not involved aside from the single meeting described above. 

Table 1 shows the sessions’ topics in detail.

The participants completed self-report questionnaires in class during the first and last session of the program. In addition, parents completed questionnaires about the child’s symptomatology at home before and immediately after the child program implementation. The program was developed in all the schools before the COVID-19 pandemic. We started it in different periods of the school year for each class (November, January, April) in order to reduce the bias related to the anxieties typical of different times of the school year.

### 2.3. Measures

#### 2.3.1. Screen for Child Anxiety-Related Emotional Disorders 

Children completed the Italian version of the 41-item Screen for Child Anxiety-Related Emotional Disorders (SCARED) questionnaire [52,53]. The SCARED questionnaire was originally devised to screen anxiety disorders in clinical samples [52,54,55,56], but it is also employed as a screening tool in community samples [56,57]. Children/mothers were asked to rate the frequency with which they/their children experienced each symptom on a 3-point Likert scale (0 = ‘almost never’, 1 = ‘sometimes’, 2 = ‘often’). Through principal component factor analysis, the authors identified five subscales [55], i.e., Panic/Somatic Anxiety (SOM), General Anxiety (GA), Separation Anxiety (SA), Social Phobia/Anxiety (SP) and School Phobia (SCH) with good internal consistency, test-rest reliability and discriminative validity. The authors reported moderate parent–child agreement (ρ = 0.20 to 0.47). 

#### 2.3.2. Child Depression Inventory 

The Child Depression Inventory (CDI) [58,59] was administered to the children to assess depression symptoms. The CDI contains 27 items, each consisting of three self-report statements graded in severity from 0 to 2, with 2 representing the severe form of a depressive symptom and 0 representing the absence of that symptom. The child was instructed to complete the CDI based on how they have been feeling during the preceding two weeks. The total score ranged from 0 to 54. The present study used the Italian version of the CDI—edited by Camuffo [59] in collaboration with Kovacs. The authors reported good psychometric properties for the Italian version of the instrument [59].

#### 2.3.3. Child Behavior Checklist/6-18

The Child Behavior Checklist/6-18 (CBCL/6-18) is a standardized questionnaire for parents to rate 113 behavioral and emotional items exhibited by their child in the past 6 months. Respondents rate each item on a 3-point Likert scale: 0 = not true; 1 = somewhat or sometimes true; and 2 = very true or often true. The 113 problem items have been factor-analyzed into eight empirically based syndrome scales [60]. The scores on the scales were calculated according to the Achenbach system of empirically based assessment (ASEBA) [60]. This system of empirically based assessment (ASEBA) [60] includes six DSM-oriented scales (DOS) aimed at covering common childhood mental disorders. The CBCL also includes three competence scales on child functioning. The competence scales assess: (1-ACTIVITIES) children’s involvement in activities (how much time they spend on sports, hobbies or games and performance compared to same-age peers; how active they are in the organizations, clubs, teams or groups to which they belong; how well they carry out jobs or chores); (2-SOCIAL) social interaction patterns (how many close friends they have, how frequently they meet with friends, how well they get along with family members and other children, how independent they are when playing or working alone); and (3-SCHOOL) school performance (performance in academic subjects, academic or other problems in school).

### 2.4. Data Analysis

Changes over time in children’s and parents’ reports on the various questionnaires were compared using pairwise *t*-tests. Given the pilot nature of this study, no adjustment was made for inflation of the Type 1 error. Analyses were performed in SPSS 26.

## 3. Results

As shown in Table 2, there were significant decreases from pre-treatment to post-treatment on all child-reported measures (all *p*’s < 0.05). In contrast, data from questionnaires completed by parents show less consistent changes. There were significant reductions over time in separation anxiety assessed by the SCARED and anxiety/depression symptoms, anxiety problems, affective problems and internalizing problems measured by CBCL. Parents also reported a significant improvement in their child’s academic functioning. However, other measures failed to show significant pre- to post-treatment change.

## 4. Discussion

The present study describes a pilot of a school-based preventive intervention for anxiety based on the Cool Kids program. The results broadly support the possible efficacy of the program in reducing symptoms of anxiety and depression, especially according to the children’s own reports. These results were partially supported by the parents’ reports.

Our findings are consistent with previous studies [26] in pointing to the efficacy of school-based programs to reduce anxiety symptoms and indicate the possible benefits of a brief format to address the core anxiety symptoms of students aged between 10 and 13 years. The study was also aimed at evaluating the efficacy of the program in reducing levels of different subtypes of anxiety symptoms. Results indicated that children who received the intervention reported improvements from pre- to post-assessment on all the SCARED subscales. Thus, from a preventive perspective, the Cool Kids Program appears to be efficacious in targeting symptoms across the domains common among different types of anxiety.

In contrast to the questionnaires completed by children, the questionnaires completed by parents did not reveal improvements in anxiety levels for most of the SCARED subscales. Anxiety symptoms are internal to the child and, consequently, may be poorly detected by other people. This may be especially the case since these children were approaching adolescence and the increased independence from family that this brings [61]. As such, the information provided by the child about their feelings, perceptions or cognitions becomes of paramount importance in the assessment process [62]. The literature has well established the tendency of parents to report fewer symptoms, suggesting that children are better informants than parents of their anxious symptoms [63,64,65,66]. In addition, the non-significant findings may be, at least in part, due to the early intervention design and the sub-clinical levels of anxiety exhibited by the majority of children. Parents may have expected to witness larger changes that are difficult to observe in subclinical children. Despite all of these difficulties, parents did report significant reductions in symptoms on all subscales of the CBCL, albeit with a smaller effect than reported by their children. Most importantly, parents reported a small but significant perceived improvement in academic functioning, pointing to a possibly important implication of the intervention. 

The results also seemed to show that the program has an effect on reducing depressive symptoms and improving academic functioning. Since the rate of comorbidity between anxiety and depression is high in children and adolescents [48], our results seem to be encouraging with respect to the reduction of this secondary measure. This is in line with previous research indicating that prevention programs for anxiety could help to prevent the development of depression in some people, with anxiety typically preceding co-morbid depressive disorders [67,68]. Our results suggest that self-reported depression symptoms are also managed by a universal anxiety-prevention program implemented in classrooms. This appears consistent with previous contributions that universal prevention interventions may potentially promote enhancement in levels of functioning in multiple problem areas [69]. 

The brief length of the program was a major strength of this study. Although the program was considerably briefer than the clinical version, these data suggest that a relatively brief intervention can demonstrate sound effects with this age group. The use of relatively brief programs has several advantages in terms of savings in costs and resources that are always difficult for many cash-strapped schools [43]. In addition, this program can be conducted efficiently within a brief timeframe, increasing the likelihood that schools would use it without compromising the regular scholastic program. 

Moreover, preventive programs in school may foster more clinically significant gains since this context provides a uniquely productive opportunity to reach a greater number of children and subclinical cases that would hardly reach clinical attention, promoting a real prevention project. The school environment also provides a real-world treatment approach and facilitates generalization [34]. Finally, collaboration with teachers also supports treatment goals. 

The results of the present study should also be considered within the context of several important limitations. Firstly, this study does not include a control group against which the outcome of the treated sample could be compared. Thus, there is no methodological control, and the observed improvement could have been attributable solely to nonspecific treatment factors (e.g., peer support, attention) or the passage of time. However, since the intervention had a very small duration and the students were not selected as high on anxiety, the positive outcomes reported here are not likely to be due to the passage of time alone. Moreover, due to the pandemic period, we could not implement a follow-up to check the maintenance of the results. Secondly, a self-report instrument was used to assess anxiety. To assess children for anxiety symptomatology, conducting a clinical interview that was designed to follow DSM–5 criteria would have been preferable. However, the literature indicated that SCARED scores have satisfactory discriminant validity (both between anxiety disorders and other problems and within anxiety disorders), and appeared to have reasonable value for predicting specific anxiety disorders [70]. Finally, the sample size was smaller than desirable and much smaller than most school-based prevention trials. However, this study still represents the first to evaluate a universal preventive program based on Cool Kids. Universal trials are realized with kids who do not necessarily present clinical diagnoses and do not seek help. They also have low scores to begin with and have little or no room to change. Therefore, the effect sizes reported in universal studies are usually very low. This means that very large samples are generally needed to show significant effects, making these current results even more impressive.

## 5. Conclusions

Although replication with a larger and more diverse sample will be necessary to clarify the utility of the intervention, Cool Kids appears to show promise as an efficacious preventive intervention for preadolescent children with anxiety symptoms. Moreover, the brevity of this intervention, coupled with its group administration in a school setting, may expand prevention efforts with children at-risk for the development of anxiety disorders (e.g., those with subclinical levels of social anxiety). Treating children at risk of developing significant anxiety symptomatology will hopefully reduce impairment in childhood as well as later in life. The possibility that academic functioning improved in this trial is especially promising in this regard. Clinical and pragmatic implications suggest that preventive interventions might foster reductions in both societal costs associated with anxiety disorders in adulthood (e.g., unemployment, welfare assistance and lost productivity) and pressures on mental health services for adults [25,71]. 

The present study provides preliminary support for the use of cognitive and behavioral techniques in treating preadolescent children with anxiety symptomatology in a preventive framework and suggests numerous avenues for future research efforts.

## Figures and Tables

**Table 1:** Intervention program components.

Session	Contents
Preliminary meeting with parents and teaching staff	Illustration of the program structure Explanation of the causes of anxiety in developmental age Explanation of the principles of the intervention and the techniques used to deal with anxiety Discussion of possible objectives to be achieved at the end of the project Proposal of functional ideas to be provided to parents and teachers for the in-depth study of the issues addressed (books, films, activities, etc.)
1. An overview of the program	Compilation of questionnaires Investigation of children’s fears through exchange and comparison Introduction to the concept of anxiety and fear Explanation of the emotional, cognitive, physiological and behavioral components of anxiety Explanation of the concept of the intensity of an emotion through the metaphor of the thermometer Comparison of the disadvantages and consequences of being controlled by one’s anxieties to increase motivation for the program Illustration of home exercises Fear mailbox setup
2. Learning to think realistically	Review of home exercises Introduction to the link between situation, thoughts, emotions and behaviors Explaining to children the difference between realistic thinking and anxious thinking Introduction to the technique of cognitive restructuring, using the metaphor of investigator thinking Group exercises on investigator thinking Illustration of the homework on investigator thinking
3. Fighting fear by facing fear	Homework review on investigator thinking Introduction to the principles of gradual exposure Beginning of creating hierarchies relevant to anxieties by building a ladder for exposure Identification of a series of prizes that children will get every time they take a step Illustration of homework relating to the execution of a few steps
4. Maintain and sustain progress	Homework review Review and construction of the stepladders Introduction to the concept of problem solving Analysis of the most common difficulties that hinder progress in the exhibition Homework on the execution of a few steps Illustration of home exercises relating to the execution of a few steps
5. Summary of the program andfinal greeting	Homework review Illustration of the concept of surfing anxiety Planning the achievement of other goals Summary of the strategies learned for managing anxiety Delivery of the diploma of courage Final compilation of the questionnaires

**Table 2:** Means, standard deviations and paired *t*-test results of Child Depression Inventory (CDI), Screen for Child Anxiety-Related Emotional Disorders (SCARED) and Child Behavior Checklist/6-18 (CBCL) for children and parents.

Rater	Scale	Pre-*M ± SD*	Post-*M ± SD*	*t*	*p*-Value
Children	CDI	10.39 ± 5.45	8.30 ± 5.66	3.622	0.001 ***
	SCARED TOT	25.74 ± 12.31	18.65 ± 11.43	4.913	0.000 ***
	SCARED SOM	6.00 ± 4.7	4.17 ± 3.93	3.353	0.001 ***
	SCARED GA	6.75 ± 3.72	5.23 ± 3.78	3.706	0.000 ***
	SCARED SA	5.19 ± 2.81	3.62 ± 2.81	4.715	0.000 ***
	SCARED SP	5.7 ± 3.45	4.26 ± 3.09	3.365	0.001 ***
	SCARED SCH	2.14 ± 1.86	1.39 ± 1.82	3.299	0.002 ***
Parents	SCARED TOT	14.85 ± 9.78	13.92 ± 9.1	0.998	322
	SCARED SOM	1.87 ± 2.56	1.79 ± 2.82	0.313	755
	SCARED GA	4.63 ± 3.03	4.53 ± 3.28	0.225	822
	SCARED SA	4.23 ± 3.37	3.55 ± 3.06	2.586	0.012 *
	SCARED SP	3.48 ± 3.09	3.32 ± 2.87	0.637	0.526
	SCARED SCH	0.65 ± 0.98	0.73 ± 0.99	−0.582	0.563
	CBCL Anxious/Depressed	55.07 ± 6.89	53.7 ± 5.01	2.109	0.039 *
	CBCL Anxiety Problems	56.74 ± 7.00	55.02 ± 6.15	2.176	0.034 *
	CBCL Internalizing	51.89 ± 9.48	49.43 ± 10.52	2.275	0.027 *
	CBCL Affective Problems	55.98 ± 7.49	54.2 ± 5.93	2.569	0.013 *
	CBCL Activities	38.43 ± 7.13	40.28 ± 9.57	−0.837	0.418
	CBCL Social	53.41 ± 5.16	52.52 ± 3.75	1.721	0.090
	CBCL School	49 ± 4.19	51.31 ± 2.6	−2.950	0.010 *

* *p* ≤ 0.05. *** *p* ≤ 0.001. TOT = Total Score; SOM = Panic/Somatic Anxiety; GA = General Anxiety; SA = Separation Anxiety; SP = Social Phobia; SCH = School Phobia.

## Data Availability

The data that support the findings of this study are available from the corresponding author, upon reasonable request.
